# Reviewed article: Lopes LPN, Raimundo ACS, Caetano R, Lopes LC. Recalculation of the budgetary impact of risperidone use for Autism Spectrum Disorder in Brazil: a case study using measured demand data, 2017-2019. Epidemiol Serv Saude. 2025:34;e20240732.

**DOI:** 10.1590/S2237-96222025v34e20240732.a

**Published:** 2025-08-08

**Authors:** Erick Soares Lisboa

**Affiliations:** Federal University of Bahia, Instituto de Saúde Coletiva

**Table:** 

Rating	Excellent	Good	Average	Below average	Poor
Interest		✓			
Quality		✓			
Originality		✓			
Overall		✓			

**Table:** 

Questionnaire	Yes	No	Not applicable
Does the manuscript contain new and significant information to justify publication?	✓		
Does the Abstract (Summary) clearly and accurately describe the content of the article?	✓		
Is the problem significant and concisely stated?	✓		
Are the methods described comprehensively?	✓		
Are the interpretations and conclusions justified by the results?	✓		
Is adequate reference made to other work in the field?	✓		

## Manuscript Structure

Length of article is: Adequate

Number of tables is: Adequate

Number of figures is: Adequate

Please state any conflict(s) of interest that you have in relation to the review of this paper (state “none” if this is not applicable): None

## Comments

Parabéns aos autores pelo trabalho, de suma relevância e importância, o artigo é excelente e recomendo para publicação com pequena sugestão: reforçar a necessidade de iniciativas nacionais para reavaliar periodicamente tecnologias incorporadas, especialmente no contexto da saúde mental e de desfinanciamento do SUS.

